# Evolutionary Conservation and Regulatory Diversification of *AS1* Homologs in Soybean

**DOI:** 10.3390/ijms262211089

**Published:** 2025-11-16

**Authors:** Dan Wang, Xuan Zhou, Dongfa Wang, Xiangtao Yang, Yexin He, Zhengjun Xia, Jianghua Chen, Weiyue Zhao

**Affiliations:** 1 CAS Key Laboratory of Tropical Plant Resources and Sustainable Use, Xishuangbanna Tropical Botanical Garden, Chinese Academy of Sciences, Kunming 650223, China; wangdan22@mails.ucas.ac.cn (D.W.);; 2 University of Chinese Academy of Sciences, Beijing 100049, China; 3 School of Life Sciences, University of Science and Technology of China, Hefei 230026, China; 4 College of Life Science, Southwest Forestry University, Kunming 650224, China; 5 State Key Laboratory of Black Soils Conservation and Utilization, Northeast Institute of Geography and Agroecology, Chinese Academy of Sciences, Harbin 150081, China

**Keywords:** leaf development, *AS1*, soybean, subfunctionalization

## Abstract

The establishment of dorsoventral polarity is a critical step in leaf morphogenesis, enabling the transition from radial primordia to flattened laminae. The MYB domain transcription factor *ASYMMETRIC LEAVES1* (*AS1*) plays a central role in this process by regulating leaf polarity and developmental transitions, primarily through the repression of Class I *KNOX* genes. Here, four *AS1* paralogs were identified in soybean (*Glycine max*), two of which showed collinearity with *Arabidopsis thaliana* and *Medicago truncatula*. The AS1 proteins of soybean and *Arabidopsis* exhibit high conservation, whereas the four *GmAS1* genes in soybean display different tissue-specific expression patterns. Strikingly, each *GmAS1* gene was able to fully rescue the defective phenotype of the *Arabidopsis as1* mutant, indicating that *GmAS1* genes are functionally conserved in leaf polarity regulation. Promoter analysis further indicated that *GmAS1* genes are enriched in *cis*-acting elements related to light response, hormone regulation, development, and stress response, suggesting potential subfunctionalization among these paralogs. In conclusion, these findings demonstrate that *GmAS1* genes are evolutionarily conserved in function but potentially diversified in regulation, providing new insights into their role in leaf polarity and stress adaptation.

## 1. Introduction

Leaf morphology plays an important role in plant growth and development by determining photosynthetic efficiency, transpiration dynamics, and resource allocation. Morphological variation influences biomass accumulation, stress adaptation, and source-sink relationships essential for reproductive success. Therefore, leaf morphology represents a key integrator of physiological performance and developmental plasticity. The establishment of leaf morphology primarily involves development along three axes: dorsoventral (adaxial–abaxial), proximal–distal (base–tip), and mediolateral (center–margin) [1]. The establishment of adaxial–abaxial polarity is particularly critical, as it transforms the leaf primordium from a radial, stem-like structure into a flattened, bilateral organ essential for physiological functions like photosynthesis and transpiration [2]. This process is controlled by a complex regulatory network of transcription factors and signaling pathways that coordinate tissue differentiation and organ morphogenesis [3].

A key component of this network is the *ARP* gene, named after *ASYMMETRIC LEAVES1* (*AS1*) from *A. thaliana*, *ROUGH SHEATH2* (*RS2*) from *Zea mays*, and *PHANTASTICA* (*PHAN*) from *Antirrhinum majus*, which belongs to a highly conserved family of MYB transcription factors [4,5,6]. Loss-of-function mutations in these genes result in severe developmental abnormalities. For example, in snapdragon, *phan* mutants produce radially symmetrical leaves that lack adaxial identity, a phenotype linked to a failure to establish proper adaxial–abaxial polarity [7]. Similarly, in maize, *rs2* mutants exhibit disorganized leaf blade–sheath boundaries and other patterning defects [5]. In *A. thaliana*, *as1* mutant leads to asymmetric, curled, or wrinkled leaves [4]. Despite these phenotypic differences, a conserved regulatory mechanism has been established: ARP proteins, in conjunction with the LATERAL ORGAN BOUNDARIES (LOB) domain protein AS2, form a repressor complex that directly binds to the promoters of Class I *KNOTTED1-like homeobox* (*KNOX1*) genes to repress their expression [4,5,6,8,9,10,11,12,13,14]. This repression prevents the retention of meristem identity in the leaf, thereby ensuring the transition from indeterminate stem cell fate to determinate leaf fate [4]. In addition to governing dorsoventral polarity in simple leaves, *ARP* gene also precisely regulate compound leaf architecture**.** The spatial domain of *PHAN* expression defines leaflet positioning along the rachis, and its restriction diminishes the adaxial identity of the leaf primordium, resulting in a morphological transition from pinnate to palmate compound leaves in *Solanum lycopersicum* [15]. These findings establish as a central regulator linking meristem maintenance with lateral organ initiation. Consistent with these conserved functions, recent research has shown that mutagenesis of *GmAS1/2* genes markedly affects leaf development and morphology in soybean [16].

Beyond its role in leaf polarity development, the *AS1* gene also functions as a developmental and environmental signal integrator, regulating floral organ abscission zones, fruit patterning, flowering time, and leaf hyponasty, while also mediating defenses against fungi, bacterial pathogens and powdery mildew [17,18,19,20,21,22,23,24]. In *M*. *truncatula*, *PHAN* also regulates petiole and rachis length, while in *S*. *lycopersicum*, *PHAN* potentially modulates leaflet initiation [15,25].

While the role of the *AS1* gene is well-established in diploid model systems like *Arabidopsis*, *A*. *majus*, and *Z*. *mays*, their evolutionary conservation and regulatory diversification in polyploid crop species remain largely unexplored. Soybean, as an ancient paleopolyploid species, is not only a cornerstone of global agriculture as the leading source of plant-derived oil and protein for food and feed, but also a key contributor to sustainable farming systems through biological nitrogen fixation. With its long history of domestication and broad adaptability, soybean holds significant economic, nutritional, and ecological value worldwide [26,27]. In addition to its agricultural significance, soybean serves as an excellent model for investigating the functional divergence of developmental regulators following gene duplication, through mechanisms such as subfunctionalization or neofunctionalization [28,29,30,31,32,33]. As leaf morphology strongly influences photosynthetic efficiency and yield, elucidating the adaptation of *AS1* genes in a complex polyploid genome is essential for bridging the gap between foundational molecular genetics and agricultural trait improvement.

To address this gap, the present study identifies four *GmAS1* genes and comprehensively analyzes their structural and functional conservation relative to *AS1*. Expression analyses showed that *GmAS1a* was preferentially expressed in shoot apices, *GmAS1b* was broadly expressed with high levels in reproductive organs and shoot apices, *GmAS1c* was predominantly expressed in leaves, and *GmAS1d* showed uniformly low expression across tissues. Functional complementation of the *Arabidopsis as1* mutant by *GmAS1* genes highlights their conserved biological function, while distinct expression patterns suggest regulatory divergence. Furthermore, *cis*-regulatory analysis of their promoters provides new evidence for regulatory diversification potentially linking developmental regulation with environmental adaptation. This work represents the first integrative characterization of the *AS1* genes in a polyploid legume, offering new insights into how gene duplication maintains essential developmental functions while enabling regulatory flexibility during crop evolution.

## 2. Results

### 2.1. Phylogenetic, Gene Structure, and Conserved Motif Analysis of AS1 Genes

**Figure 1:**
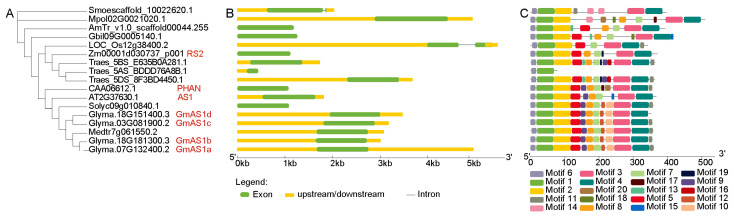
Evolutionary analysis, gene structures, and conserved motifs of *AS1* genes in twelve representative species. (**A**) Maximum-likelihood (ML) phylogenetic tree of AS1 constructed using MAFFT and IQ-TREE with 1000 bootstrap replicates. Genes highlighted in red indicate the four GmAS1 proteins and the previously characterized AS1 from *Arabidopsis*, RS2 from *Antirrhinum*, and PHAN from maize. (**B**) Exon–intron structures of *AS1* genes. The green rectangles represent exons, the yellow boxes indicate upstream/downstream regions, and black lines represent introns. (**C**) Conserved motif composition of AS1 proteins. Twenty distinct motifs are shown as colored boxes. Motif sequences are shown in Figure S1.

To elucidate the evolutionary relationships of the *AS1* gene across plant lineages, twelve representative species were selected, spanning *Bryophyta* (*Marchantia polymorpha*), *Pteridophyta* (*Selaginella moellendorffii*), gymnosperms (*Ginkgo biloba*), basal angiosperms (*Amborella trichopoda*), and diverse angiosperms such as *A. thaliana*, *A. majus*, *M. truncatula*, *G. max*, *S. lycopersicum*, *Z. mays*, *Triticum aestivum* and *Oryza sativa*. Phylogenetic analysis revealed that the *AS1* gene is absent in algae but present in both mosses and ferns (Figure 1A). This distribution pattern indicates that *AS1* likely originated early in land plant evolution, after their divergence from algal ancestors. Its conservation across the land plant lineages suggests *AS1* plays a fundamental role in the development and morphological differentiation of land plants. The retention of a single-copy *AS1* gene in most of these lineages further implies strong evolutionary constraints, possibly due to its involvement in essential regulatory pathways critical for adaptation to terrestrial environments. In contrast, multiple copies of *AS1* were identified in soybean and wheat, with four genes in soybean which are designated as *GmAS1a* (Glyma.07G132400), *GmAS1b* (Glyma.18G181300), *GmAS1c* (Glyma.03G081900), and *GmAS1d* (Glyma.18G151400), respectively [16].

Analysis of *AS1* gene structures revealed notable variation among species, ranging from single-exon genes without UTRs to multi-exon forms with both 5′ and 3′ UTRs (Figure 1B). Despite these structural differences, AS1 proteins were highly conserved, with Motifs 6 and 1 present across all species, while a few motifs showed lineage-specific loss (Figure 1C). In conclusion, these findings indicate that although the structure and motif composition of *AS1* have diversified across plant lineages, several motifs remain highly conserved, reflecting their critical role in the evolution and functional maintenance of AS1 proteins.

### 2.2. Collinearity Analysis of the AS1 Genes Among A. thaliana, M. truncatula, and G. max

**Figure 2:**
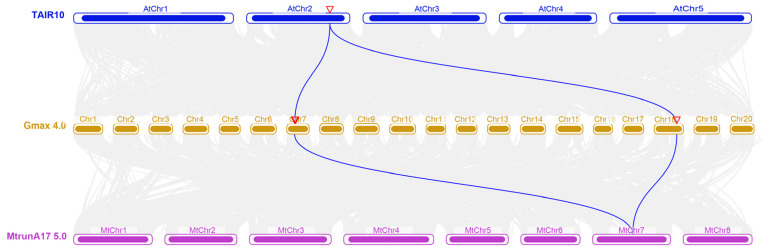
The collinearity analysis of *AS1* genes among *A. thaliana*, *G. max*, and *M. truncatula*. The *AS1* gene on *Arabidopsis* chromosome 2 (marked by a red inverted triangle) is collinear with the *AS1* genes on soybean chromosomes 7 and 18, whereas the *AS1* gene on *M. truncatula* chromosome 7 also exhibits collinearity with the *AS1* genes on soybean chromosomes 7 and 18.

The *AS1* gene is typically present as a single copy in most plant species; but four copies were identified in *G. max*, likely due to two rounds of whole-genome duplication events in its evolutionary history. To trace their origins, collinearity analyses were conducted among *A. thaliana*, *M. truncatula*, and *G. max*. The results showed that only *GmAS1a* and *GmAS1b* retained syntenic relationships with *A. thaliana* and *M. truncatula* (Figure 2). This finding suggests that these two copies were derived from ancestral loci conserved through polyploidization, whereas the *GmAS1c* and *GmAS1d* likely originated from subsequent segmental duplications or chromosomal rearrangements, resulting in the loss of detectable synteny.

### 2.3. Sequence Conservation Among AS1 Proteins in Arabidopsis and Soybean

**Figure 3:**
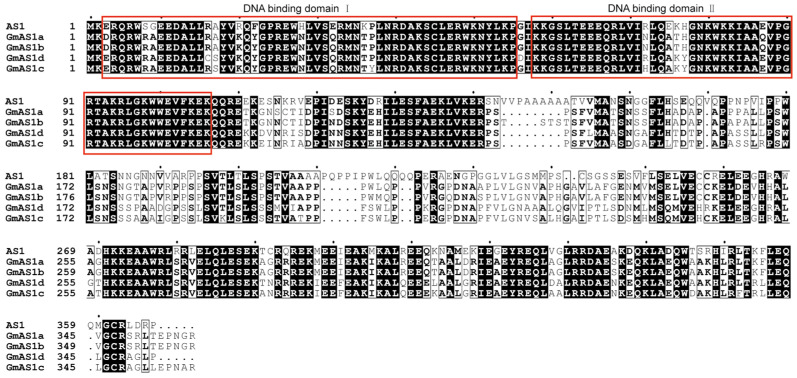
Sequence alignment and conserved domain of AS1 homologous proteins. Multiple sequence alignment of AS1 homologous proteins from *A. thaliana* and *G. max*. Conserved residues are shaded in black, and red boxes indicate DNA-binding domains I and II.

Multiple sequence alignment of *Arabidopsis* AS1 and the four soybean homologs revealed a high degree of amino acid conservation (Figure 3). Two distinct DNA-binding domains were clearly identified, showing near-identical sequences across all five proteins, indicating strong evolutionary constraint on these regions. In contrast, the C-terminal portions exhibited pronounced sequence divergence, suggesting potential diversification in regulatory roles or protein–protein interaction.

The sequence logo analysis further supported these observations (Figure S2). Consistent with the alignment, residues within the DNA-binding domains were highly conserved, displaying strong positional constraints and minimal substitutions, underscoring their essential function in DNA recognition and transcriptional regulation. In conclusion, these findings indicate that while the N-terminal and central domains of AS1 proteins are strongly conserved and functionally indispensable, the C-terminal region exhibits greater sequence plasticity, potentially contributing to functional diversification among soybean *GmAS1* paralogs.

### 2.4. Expression Pattern of GmAS1 Genes

**Figure 4:**
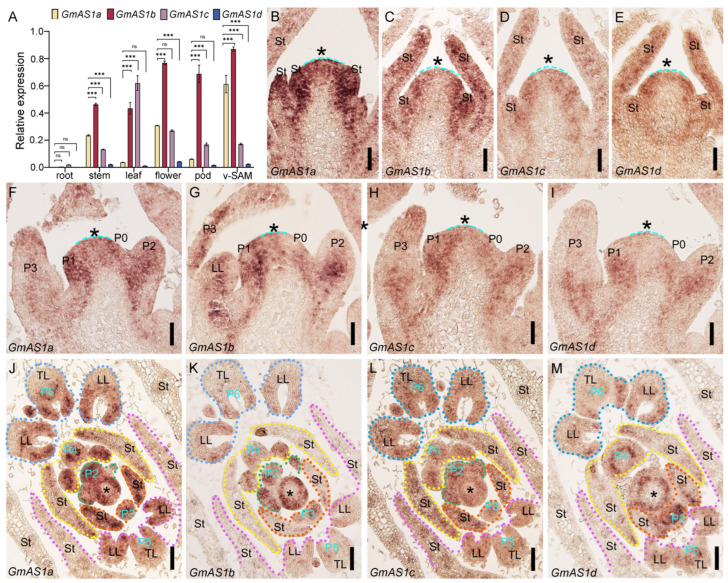
Expression pattern of *GmAS1* genes in different tissues and shoot apices. (**A**) qRT–PCR analysis showing relative expression levels of *GmAS1a–d* in roots, stems, leaves, flowers, pods, and vegetative shoot apical meristems (v-SAM). The housekeeping gene *GmACTIN-11* was used as the internal reference. Data are shown as mean ± SD (*n* = 3). Statistical significance was evaluated by two-way ANOVA (*** *p* < 0.001, extremely significant; ns, not significant). Comparable results were consistently observed in three independent biological replicates. (**B**–**I**) In situ hybridization showing the spatial expression of *GmAS1a* (**B**,**F**), *GmAS1b* (**C**,**G**), *GmAS1c* (**D**,**H**), *GmAS1d* (**E**,**I**) in shoot apices, with transcripts accumulated in the shoot apical meristem (asterisks), young leaf primordia (P0–P3), and stipule (St). (**J**–**M**) Transverse sections of developing leaf primordia showing *GmAS1* expression in stipules, terminal leaflets (TL), and lateral leaflets (LL). P, plastochron; St, stipule; TL, terminal leaflet. LL, lateral leaflet. Scale bars indicate 50 μm (**B**–**I**) and 100 μm (**J**–**M**).

To characterize the spatial expression divergence among *GmAS1* paralogs, qRT-PCR analysis was performed to quantify their transcript levels in roots, stems, leaves, flowers, pods, and vegetative shoot apices. The four *GmAS1* genes exhibited distinct tissue-specific expression patterns (Figure 4A). *GmAS1a* was most strongly expressed in the vegetative shoot apices, with lower levels in stems and flowers. *GmAS1b* was broadly and highly expressed, with the highest levels in flowers, pods, and the vegetative shoot apices, moderate expression in stems and leaves, and weak expression in roots. *GmAS1c* was mainly expressed in leaves, with moderate levels in flowers. *GmAS1d* exhibited uniformly low expression across all tissues. Collectively, these patterns suggest subfunctionalization, with *GmAS1a* specialized in the vegetative shoot apices, *GmAS1b* broadly active, and *GmAS1c* associated with leaf development, while *GmAS1d* shows no strong tissue preference, possibly undergoing functional redundancy or silencing.

Consistent with these quantitative data, RNA in situ hybridization further confirmed the spatial expression pattern of the four *GmAS1* genes. Their transcripts were mainly detected in the developing leaf primordia, young leaves, stipules, and stem vascular bundles (Figure 4B–I). Transverse sections further corroborated their accumulation in these tissues (Figure 4J–M).

### 2.5. Genetic Complementation Analysis

**Figure 5:**
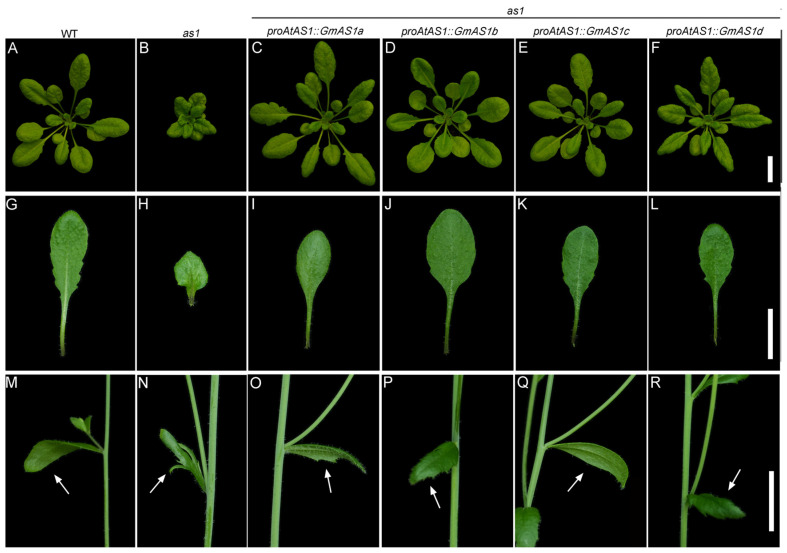
Functional complementation of the *Arabidopsis as1* mutant by *GmAS1* genes. (**A**–**F**) Rosette phenotypes of wild type (WT), *as1* mutant, and *as1* mutant expressing *GmAS1a–d* under the control of the *AtAS1* promoter (*proAtAS1::GmAS1a–d*). Expression of each *GmAS1* gene restored the flat, expanded leaf morphology of the *as1* mutant. (**G**–**L**) The *as1* mutant produced small, upward-curled leaves, whereas *proAtAS1::GmAS1a–d* lines exhibited a nearly wild-type lamina shape. (**M**–**R**) Cauline leaves subtending axillary branches. The *as1* mutant produced deeply lobed cauline leaves with fused petioles and laminae (arrows), whereas expression of *GmAS1a–d* under the *AtAS1* promoter (*proAtAS1::GmAS1a–d*) restored the simple, entire cauline leaf morphology characteristic of the wild type. Bar = 2 cm.

To evaluate the function conservation of *AS1* between soybean and *Arabidopsis*, the coding sequences of *GmAS1a*, *GmAS1b*, *GmAS1c*, and *GmAS1d* were individually driven by the *Arabidopsis AS1* promoter and introduced into the *as1* mutant. PCR-based genotyping verified the mutant background and confirmed the presence of each *GmAS1* transgene, and no fewer than three independent complementation lines were obtained for each construct. Phenotypic analyses showed that expression of each of the four soybean *AS1* paralogs restored wild-type leaf morphology (Figure 5, Figures S3 and S4). These results demonstrate that the core developmental function of *AS1* is evolutionarily conserved between soybean and *Arabidopsis*. Moreover, the capacity of all four soybean paralogs to complement the *as1* mutant highlights their high degree of functional conservation, suggesting strong evolutionary constraints that have preserved *GmAS1* function following duplication.

### 2.6. Prediction of Cis-Acting Elements

**Figure 6:**
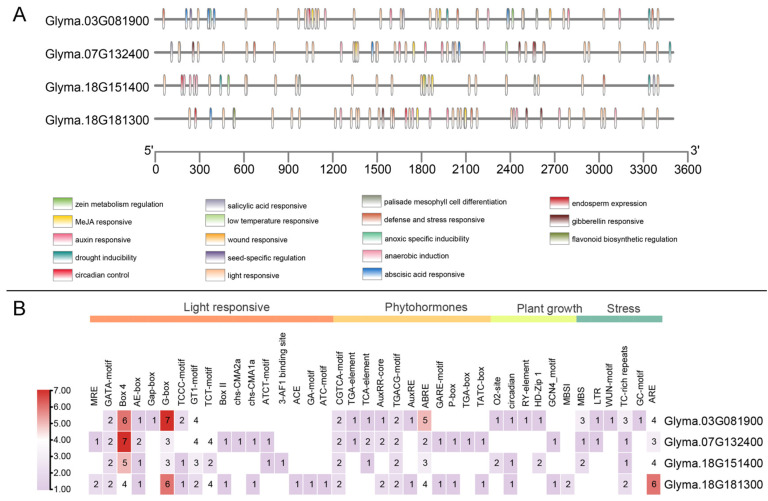
*Cis*-acting regulatory elements in the promoter regions of soybean *AS1* homologs. (**A**) Distribution of predicted *cis*-elements within the 3.5 kb upstream regions of four *AS1* homologs. Elements are color-coded according to their putative functions, including responses to light, phytohormones, stress, and developmental processes. (**B**) Heatmap showing the frequency of each *cis*-element type in the four promoters. Categories include light-responsive, phytohormone-related, plant growth, and stress-related elements.

Analysis of the 3.5 kb promoter region upstream of the *GmAS1* genes start codon using the PlantCARE database revealed a dense distribution of *cis*-acting elements associated with light response, hormone signaling, growth and development, and stress response (Figure 6). Light-responsive elements were the most abundant, with 18 core motifs identified, including MRE, GATA-motif, Box 4, AE-box, gap-box, and G-box, suggesting that the expression of *GmAS1* genes is tightly regulated by light signaling networks. The promoter also contains multiple hormone-responsive elements, including jasmonic acid (CGTCA-motif, TGACG-motif), auxin (AuxRR-core, TGA-element), gibberellin (GARE-motif, P-box), and abscisic acid (ABRE) response elements, among a total of 12 types, indicating potential regulation by multiple phytohormones. In addition, six elements related to growth and development processes were identified, such as O2-site (endosperm-specific expression), circadian (circadian rhythm regulation), and RY-element (seed development regulation), indicating possible roles in specific developmental stages or tissues. Notably, the promoter contains six types of stress-responsive elements, including MBS (drought), LTR (cold), WUN-motif (wounding), TC-rich repeats (defense and stress response), GC-motif (hypoxia), and ARE (anaerobic induction). Based on the reported regulatory roles of MYB transcription factors in biotic and abiotic stress responses [34], these findings suggest that *GmAS1* genes may play a key role in regulating soybean responses to abiotic stresses such as drought and low temperature.

## 3. Discussion

The soybean genome, shaped by multiple duplication events, provides an excellent model for investigating the evolution of developmental genes. As an ancient paleopolyploid, soybean has undergone at least two rounds of whole-genome duplication (WGD), the most recent occurring approximately 13 million years ago [32]. Despite extensive gene loss following these events, about 70–75% of soybean genes remain in duplicated copies [32,33]. This extensive genomic redundancy facilitated the expansion of the *GmAS1* gene, yielding four homologs that have likely undergone subfunctionalization and regulatory diversification after duplication [35]. This trend of post-duplication divergence is not merely transcriptional but extends to the underlying regulatory mechanisms, and is heavily influenced by the mode of gene duplication. While large-scale expression profiling revealed that a majority of duplicated gene pairs display divergent expression patterns [36], further analysis shows that WGD-derived genes are significantly more prone to divergence through *trans*-regulatory changes, whereas small-scale duplicates (e.g., singletons, tandem) show a higher proportion of *cis*-regulatory divergence [37].

In this study, we systematically analyzed four *GmAS1* paralogs, combining phylogenetic, structural, expression, and functional analyses to elucidate their evolutionary conservation and potential regulatory divergence. Our results reveal that, while all four *GmAS1* paralogs retain highly conserved protein domains and developmental functions, they have undergone regulatory diversification reflected by distinct tissue-specific expression profiles and promoter compositions. These findings provide novel insights into how gene duplication in polyploid crops promotes the balance between evolutionary constraint and regulatory innovation.

### 3.1. Evolutionary Conservation of AS1 Function

Comparative phylogenetic and sequence analyses demonstrated that *AS1* homologs are deeply conserved across land plants, from bryophytes to angiosperms, indicating its early establishment as a fundamental developmental regulator. The strong conservation of two MYB DNA-binding domains across soybean and *Arabidopsis* AS1 proteins suggests that evolutionary constraints have maintained these regions to safeguard their molecular function. Consistent with this, all four *GmAS1* paralogs successfully complemented the *Arabidopsis as1* mutant phenotype, restoring nearly wild-type leaf morphology. These results underscore that the core molecular function of *AS1*-mediating *KNOX* gene repression to maintain leaf determinacy-is evolutionarily stable.

Such evolutionary stability parallels findings from other species, including *Selaginella kraussiana*, *Antirrhinum*, *Z. mays* and *Cardamine hirsute*, where *AS1* orthologs can similarly rescue *as1* phenotypes despite distinct leaf morphologies across species [1,12,38]. Together, these results underscore that morphological diversity across taxa is unlikely to result from changes in AS1 protein function itself, but rather from variation in its regulatory networks. This observation sets the stage for understanding how duplicated *AS1* genes in soybean maintain their conserved function while evolving divergent transcriptional regulation.

### 3.2. Regulatory Diversification of GmAS1 Paralogs

Despite their conserved protein domains and functional redundancy in *Arabidopsis*, the four *GmAS1* genes display distinct expression profiles, indicating regulatory divergence following duplication. *GmAS1a* is preferentially expressed in vegetative shoot apices, *GmAS1b* exhibited broad expression across both reproductive and vegetative tissues, and *GmAS1c* showed leaf-enriched expression, while *GmAS1d* maintains low transcript levels across tissues. These complementary expression domains suggest subfunctionalization, where duplicated genes partition ancestral regulatory functions to avoid redundancy and optimize developmental precision [39,40].

Promoter analysis further supports this notion. Notably, six types of stress-responsive *cis*-elements were detected in the *GmAS1* promoters, including MBS (drought), LTR (cold), WUN-motif (wounding), TC-rich repeats (defense and stress response), GC-motif (hypoxia), and ARE (anaerobic induction). The presence of stress-related *cis*-elements indicates the potential involvement of *GmAS1* in defense-related signaling pathways, consistent with the reported role of *AS1* in mediating defenses against fungi, bacterial pathogens, and powdery mildew. Moreover, the variation in motif composition among paralogs points to divergent *cis*-regulatory architectures, which may enable individual *GmAS1* copies to respond to distinct physiological or environmental cues. Such diversification likely provides a flexible transcriptional framework for integrating developmental and immune signaling, thereby fine-tuning growth-defense balance in soybean.

### 3.3. Evolutionary and Agronomic Implications

From an evolutionary perspective, the retention of four functionally conserved yet differentially regulated *AS1* paralogs in soybean exemplifies how gene duplication promotes the balance between functional constraint and regulatory innovation. Such retention likely confers developmental robustness, ensuring stable leaf polarity and morphology, while allowing flexible responses to environmental or hormonal cues. This duality reflects a broader evolutionary strategy in polyploid plants, where duplicated developmental regulators are retained not for novel biochemical roles, but for expanded regulatory capacity.

Agronomically, elucidating the regulatory diversification of *AS1* paralogs offers valuable insights for soybean improvement. Leaf architecture directly determines photosynthetic efficiency and yield potential. The ability to manipulate *GmAS1* expression patterns through targeted *cis*-regulatory modifications could enable precise control of leaf form and stress adaptability without disrupting essential developmental pathways. Given its role in coordinating morphogenetic and defense pathways, fine-tuning the expression of specific *GmAS1* copies may further enhance stress resilience while maintaining optimal growth and development. Future studies integrating genome editing, chromatin accessibility profiling, and environmental transcriptomics will be critical to unravel how regulatory variation in AS1 networks contributes to phenotypic plasticity in polyploid crops.

## 4. Materials and Methods

### 4.1. Plant Materials and Growth Conditions

In this study, the *as1* mutant (SALK_210101C) was obtained from AraShare (https://www.arashare.cn/index/), with *Arabidopsis* Col-0 and the soybean cultivar Williams 82 serving as wild-types. All plants were cultivated under long-day conditions (LD, 16 h light/8 h dark) in a temperature-controlled greenhouse maintained at 22 °C. All primer sequences are listed in Table S1.

### 4.2. Identification of AS1 Genes in Plant Species

To elucidate the evolutionary relationships of the *AS1* gene across plant lineages, twelve representative species were selected for evolutionary analysis, encompassing *M. polymorpha*, *S. moellendorffii*, *G. biloba*, *A. trichopoda*, *A. thaliana*, *A. majus*, *M. truncatula*, *G. max*, *S. lycopersicum*, *Z. mays*, *T. aestivum*, and *O. sativa*. The *Arabidopsis* AS1 and maize RS2 protein sequences were used as queries in BLAST searches against Phytozome v14 (https://phytozome-next.jgi.doe.gov/), the PGCP database (https://biobigdata.nju.edu.cn/pgdatabase/home), and NCBI (https://www.ncbi.nlm.nih.gov/) to identify and retrieve homologous *AS1* gene and protein sequences from these species (accessed on 12 August 2025) [41].

### 4.3. Phylogenetic, Gene Structure, and Conserved Motif Analysis of AS1 Homologs

Multiple sequence alignment of identified AS1 protein homologs was conducted using MAFFT with default parameters [42]. Phylogenetic trees were subsequently constructed using the maximum likelihood (ML) method implemented in IQ-TREE [43]. The resulting trees were subsequently refined and visualized using iTOL [44]. Gene structures were illustrated using GSDS 2.0 (https://gsds.gao-lab.org/). Conserved motifs were identified with MEME (https://meme-suite.org/meme/tools/meme (accessed on 12 August 2025)), and the resulting file was visualized by TBtools (v2.327) [45].

### 4.4. Collinearity Analysis of the AS1 Genes

The genome sequences (fasta) and annotation features (gff) of *A. thaliana* (TAIR 10), *Glycine max* (v4.0) and *M. truncatula* (A17 r5.0) were obtained from NCBI collinearity analyses between *A. thaliana* and *G. max*, as well as between *G. max* and *M. sativa*, were conducted using One Step MCScanX module in TBtools, and these results were subsequently integrated with the Text Merge for MCScanX function in TBtools.

### 4.5. Mutiple Sequence Alignment of AS1 Homologs

To investigate the sequence conservation of AS1 proteins between *A. thaliana* and *G. max*, protein sequences of AtAS1, GmAS1a, GmAS1b, GmAS1c, and GmAS1d were aligned using Clustal Omega (https://www.ebi.ac.uk/jdispatcher/msa/clustalo?stype=protein (accessed on 15 February 2025)) with default parameters. The resulting alignment was visualized and formatted with ESPript 3.0 (https://espript.ibcp.fr/ESPript/ESPript/ (accessed on 15 February 2025)). To further assess residue conservation across the five proteins, WebLogo 3.7.9 (https://weblogo.threeplusone.com/) was employed to generate sequence logos, providing a graphical representation of positional amino acid frequencies. Conserved domains and functional motifs were identified using the SMART database (https://smart.embl.de/), which guided domain annotation in the alignment and sequence logo analyses.

### 4.6. RNA Extraction and Quantitative RT-PCR (qRT-PCR) Analysis

Vegetative shoot apices, roots, stems, and leaves of Williams 82 at 21 days after germination, as well as flowers and pods at the reproductive stage, were collected for total RNA extraction. First-strand cDNA was synthesized from 2 μg total RNA using a HiScriptII 1st Strand cDNA Synthesis Kit (Vazyme Biotech, Nanjing, China). qRT-PCR was performed using a Roche LightCycler 480 system (Roche Diagnostics International Ltd., Rotkreuz, Switzerland) in 10 μL reaction volumes containing 5 μL of 2× Quantfast Green qPCR SuperMix (Maibo Biotech, Hangzhou, China), 0.3 μL of specific primers, 0.2 μL of 50× Rox Dye, 1 μL of 1:5 dilution of reverse transcription product, and 3.2 μL of dd H_2_O. The relative expression levels of each gene were calculated by the 2^−ΔCT^ method using the housekeeping gene *GmACTIN-11* (Glyma.18G290800) as the reference gene [46,47].

### 4.7. RNA in Situ Hybridization

Full-length CDS with 5′ UTR of *GmAS1a*, *GmAS1b*, *GmAS1c*, *GmAS1d* served as templates to synthesize digoxigenin-labeled antisense RNA probes. Three-week-old seedling vegetative shoot apices were sectioned into eight 8-μm-thick serial sections and hybridized with the probes as previously described [48]. The signals were observed using an Olympus BX63 microscope (Olympus, Tokyo, Japan) with differential interference contrast (DIC) imaging.

### 4.8. Genetic Complementation Analysis

The promoter region of *AtAS1* (*proAtAS*, 2000 bp) and the full-length coding sequences (CDS) of *GmAS1a* (1086 bp), *GmAS1b* (1074 bp), *GmAS1c* (1074 bp), and *GmAS1d* (1059 bp) were amplified by PCR with Phanta Super-Fidelity DNA Polymerase (Vazyme Biotech, Nanjing, China). The CDS of *GmAS1a*, *GmAS1b*, *GmAS1c* and *GmAS1d* were individually fused by overlapping PCR to the *proAtAS1*, and subsequently constructed into the *pCAMBIA3301-NM* vector digested with *BamH I* and *Pml I* endonucleases. The recombinant plasmids were verified by Sanger sequencing to ensure sequence integrity and correct insertion orientation. Subsequently, the validated constructs were introduced into *Agrobacterium tumefaciens* strain EHA105 cells. Following bacterial colony PCR verification, the transformed *Agrobacterium* cultures were used for floral dip transformation of *Arabidopsis thaliana as1* mutant plants [49]. Transformed seeds were sown in nutrient soil and were selected by three sequential spray applications of 0.002% Basta solution at the first true-leaf stage. Herbicide-surviving plants with wild-type morphology were genotyped by PCR to confirm transgene integration.

### 4.9. Prediction of Cis-Acting Elements

Putative promoter regions (3.5 kb upstream of the start codon) of *GmAS1* genes were retrieved from the Phytozome v14 database. These sequences were subsequently analyzed for *cis*-regulatory elements using PlantCARE (https://bioinformatics.psb.ugent.be/webtools/plantcare/html/ (accessed on 15 February 2025)). Motif identification and visualization were subsequently conducted in TBtools.

## 5. Conclusions

Collectively, this study demonstrates that duplicated *AS1* genes in soybean maintain conserved protein function but diversified regulation, illustrating how polyploidy drives regulatory evolution while safeguarding core developmental mechanisms. This work provides a framework for linking gene duplication to morphological innovation and environmental adaptation in complex crop genomes.

## Data Availability

Data is contained within the article or Supplementary Materials. The original contributions presented in this study are included in the article/Supplementary Materials. Further inquiries can be directed to the corresponding authors.

## Supplementary Materials

The following supporting information can be downloaded at https://www.mdpi.com/article/10.3390/ijms262211089/s1.
