# Supplementary material for: Evolutionary Conservation and Regulatory Diversification of AS1 Homologs in Soybean
Source: Int J Mol Sci. 2025 Nov 16;26(22):11089. doi: 10.3390/ijms262211089 (PMC12652363; doi:10.3390/ijms262211089)
Supplement: Supplementary file 1 [file ijms-26-11089-s001.zip › Figure S1-S4.pdf]

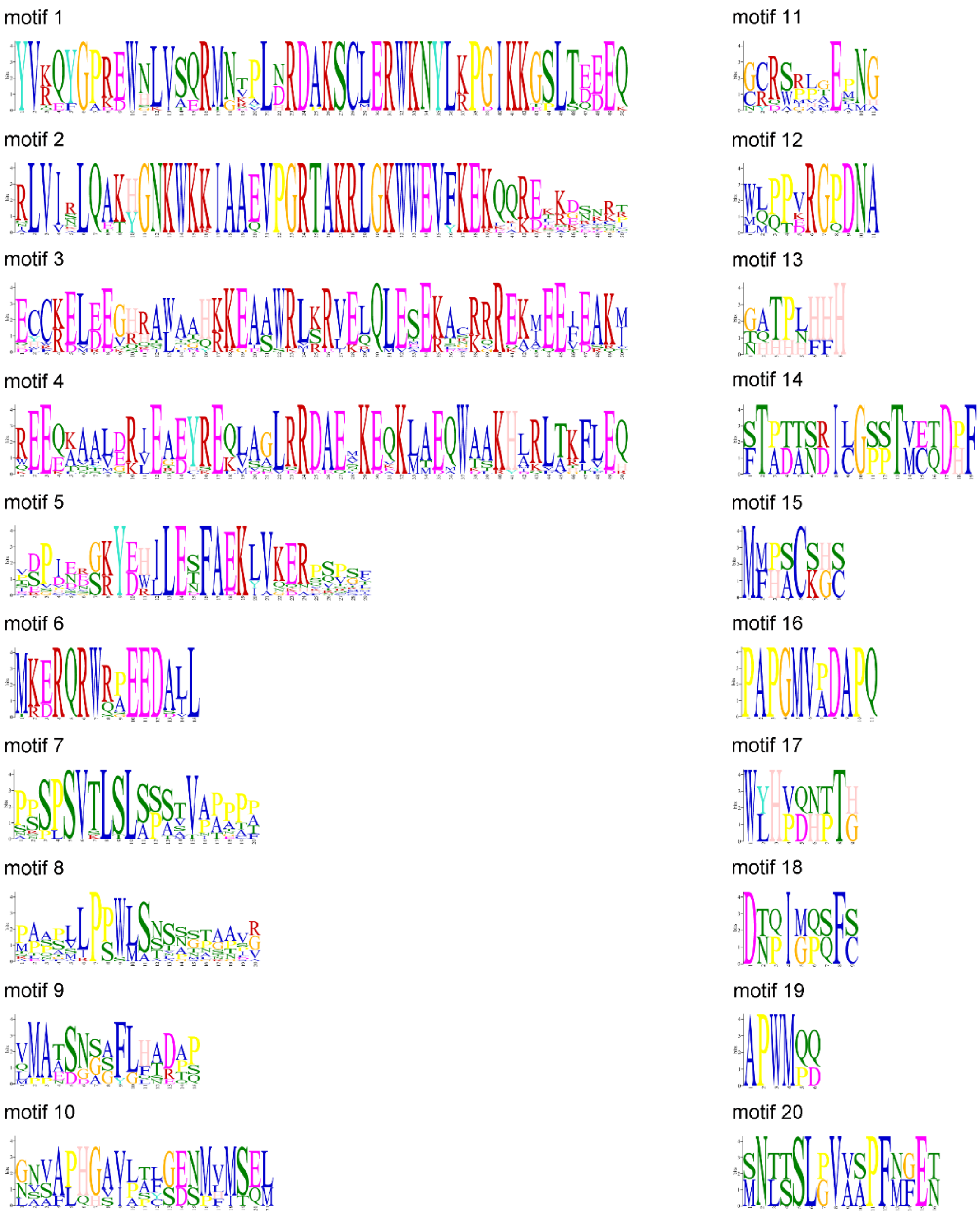

Figure S1. Consensus sequence logos of 20 conserved motifs identified in the GmAS1 proteins.

The overall height of the stack of letters indicates the conservation of the motif, while the height of each letter within the stack represents the relative frequency of that amino acid at that position. The analysis was performed using MEME Suite with the full-length protein sequences.

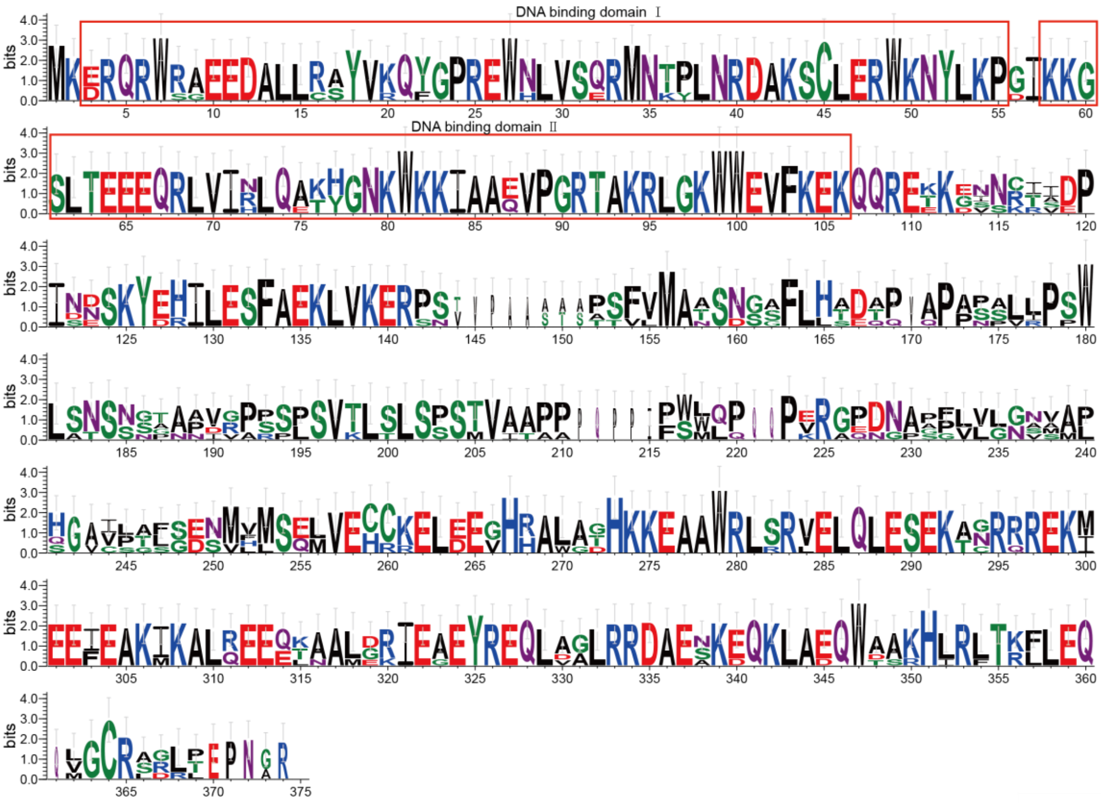

Figure S2. Sequence alignment and conserved domain of AS1 homologous proteins. Sequence logo representation of conserved residues, with letter height reflecting conservation. DNA-binding domains are highlighted in red boxes.

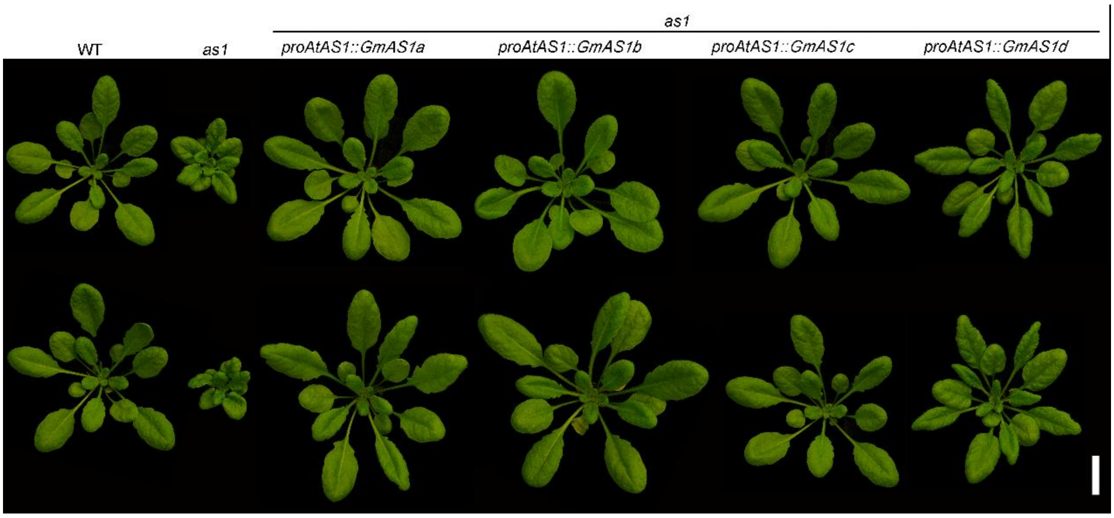

Figure S3. Functional complementation of the *Arabidopsis as1* mutant by *GmAS1* genes. Rosette phenotypes of wild type (WT), *as1* mutant, and *as1* mutant expressing *GmAS1a-d* under the control of the *AtAS1* promoter (*proAtAS1::GmAS1a-d*). Expression of each *GmAS1* gene restored the flat, expanded leaf morphology of the *as1* mutant. Bar = 2 cm.

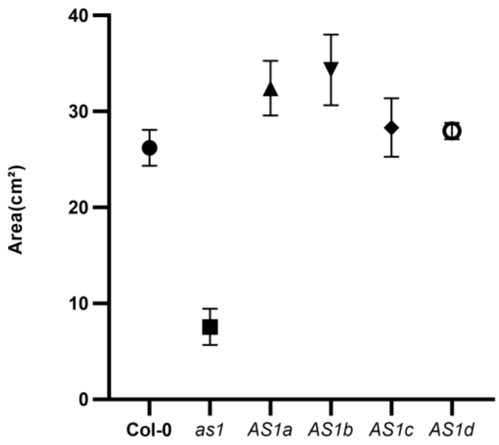

Figure S4. Leaf area of *Arabidopsis* wild-type, *as1* mutant, and transgenic *as1* plants expressing *GmAS1* genes.

Quantitative analysis of leaf area (cm^2) in 40-day-old rosette leaves of *Arabidopsis* Col-0 (wild type), *as1* mutant, and *as1* mutant lines expressing *GmAS1a–d*. Data represent means ± SD (n = 3 biological replicates).
